# Effectiveness and clinical benefits of new anti-diabetic drugs: A real life experience

**DOI:** 10.1515/med-2022-0504

**Published:** 2022-07-07

**Authors:** Giuseppina Piazzolla, Alfredo Vozza, Sara Volpe, Alessandro Bergamasco, Vincenzo Triggiani, Giuseppe Lisco, Michela Falconieri, Cosimo Tortorella, Vincenzo Solfrizzi, Carlo Sabbà

**Affiliations:** Interdisciplinary Department of Medicine, University of Bari “Aldo Moro,” School of Medicine, Bari, Piazza G. Cesare 11, 70124 Bari, Italy; Interdisciplinary Department of Medicine, University of Bari “Aldo Moro,” School of Medicine, Bari, 70124 Bari, Italy

**Keywords:** SGLT2i, GLP1RA, Type 2 diabetes, C-peptide, insulin therapy

## Abstract

We evaluated the clinical impact, in daily clinical practice, of sodium-glucose co-transporter-2 inhibitors (SGLT2i) and glucagon-like peptide-1 receptor agonists (GLP1RA) therapies in patients with type 2 diabetes. Data from 500 unselected consecutive patients were retrospectively analyzed. Only those with a full assessment at baseline (T0) and after 3 (T3), 6 (T6), and 12 (T12) months of treatment with SGLT2i or GLP1RA were included in the study (*n* = 167). At baseline, patients had a high mean body weight (BW), abdominal circumference (AC), body mass index (BMI), and HOMA index. Despite normal C-peptide values, 39 patients were being treated with insulin (up to 120 IU/day). During therapy, a progressive improvement in BW, BMI, and AC was observed with both the molecules. Fasting glucose and glycated Hb decrease was already significant at T3 in all patients, while the HOMA index selectively improved with SGLT2i therapy. Renal function parameters remained stable regardless of the drug used. Finally, SGLT2i reduced serum uric acid and improved the lipid profile, while GLP1RA reduced serum levels of liver enzymes. Both the therapeutic regimens allowed a significant reduction or complete suspension of unnecessary insulin therapies. Our real life data confirm the results obtained from randomized clinical trials and should be taken as a warning against inappropriate use of insulin in patients with preserved β-cell function.

## 1 Introduction

Type 2 diabetes (T2D) is considered a global health emergency, mainly due to its high prevalence. Indeed, according to the most recent epidemiological estimates of the International Diabetes Federation, the prevalence is expected to double over the next 25 years [1]. These figures may be ascribed to population aging, earlier diagnosis, and the increased survival of diabetic patients thanks to improvements in the quality of care and recent advances in the pharmacological field. Undoubtedly, the therapeutic management of T2D has undergone radical and revolutionary changes in recent years. The new therapeutic targets, in fact, are no longer simply glycemic control, the reduction in glycated Hb (HbA1c), or minimization of the hypoglycemic risk. Instead, they are now increasingly oriented toward reducing the cardiovascular risk (CVR) and the overall mortality of diabetic patients, thus moving away from a glucocentric toward a cardio-metabolic approach [2,3,4]. Such ambitious goals are currently achievable thanks to the proven nephron- and cardio-protection provided by two classes of new antidiabetic drugs, i.e., sodium-glucose co-transporter-2 inhibitors (SGLT2i) and glucagon-like peptide-1 receptor agonists (GLP1RA) [3,4]. The numerous clinical trials published in the past years highlight a broad spectrum of beneficial effects with both the drug classes, ultimately improving the outcome of diabetic disease complications. As known, however, randomized clinical trials (RCTs) enroll selected populations of subjects and create optimal conditions for achieving the desired results [5,6]. These goals are much more difficult to achieve in clinical practice, especially when dealing with patients with co-morbidities [7]. In this context, real life clinical studies are particularly useful to validate data obtained in the more artificial RCT environments and to understand how efficacious a drug really is.

The purpose of this study was to evaluate the clinical impact of SGLT2i or GLP1RA on T2D management in the context of normal clinical practice, when dealing with all the variables of everyday life. To this aim, data were collected retrospectively from a population of T2D outpatients. Patients who had been treated with one of these two classes of antidiabetic agents and having a full 1 year follow-up available, were included in the study and their baseline characteristics were analyzed and compared. The effects of SGLT2i or GLP1RA on patient biometric parameters and laboratory tests were then studied.

## 2 Patients and methods

### 2.1 Study population

In this observational retrospective study, we analyzed data from 500 consecutive T2D patients (331 males and 169 females, mean age of 63.8 ± 10.4 years), attending the Metabolic Disorders Outpatients Clinic of the Department of Internal Medicine, at the University of Bari Medical Center, from June 2018 to July 2020. Exclusion criteria were any kind of cancer within less than 5 years prior to the study, infections or systemic corticosteroid treatment in the last 4 weeks, diagnosis of type 1 diabetes, and previous use of a drug belonging to one of the two classes under study. To be included in the study, patients had to be diagnosed with T2D. Moreover, after the complete evaluation of their clinical, bio-humoral, and instrumental picture, they had to have received the prescription of a SGLT2i or a GLP1RA. Only patients who had undergone a full outpatient assessment at baseline (T0) and after 3 (T3), 6 (T6), and 12 (T12) months of therapy with one of the two drugs were finally included in the present study (*n* = 167, 113 males and 54 females, mean age 62.9 ± 9.5 years). At all observations, the general visit included measurements of height, body weight (BW), body mass index (BMI), abdominal circumference (AC), systolic (SBP) and diastolic blood pressure (DBP), and heart rate (HR), as well as venous sampling for routine tests, including serum uric acid, vitamin D, HbA1c, C-peptide, and insulin after a 12 h fasting period. The HOMA index was calculated as (fasting insulin × fasting glucose)/405 (normal range 0.23–2.5). All medications taken by the patients at the time of inclusion in the study were recorded, paying particular attention to metformin, oral antidiabetics, short-acting (SA) and/or long-acting (LA) insulin analogues. Patients were subdivided into group 1 (*n* = 54, 37 males and 17 females) and group 2 (*n* = 113, 76 males and 37 females), depending on whether the drug prescribed at T0 was a SGLT2i or a GLP1RA, respectively. It should be noted that the simultaneous administration of the 2 classes of drugs was not reimbursed by the Italian National Health System during the entire study period. Consequently, since only few patients agreed to buy one of the 2 drugs for use in combination, these patients’ data were not included in the study. In group 1, 21 patients had been treated with empagliflozin, 18 with dapagliflozin, and 15 with canagliflozin. In group 2, 8 patients had been treated with daily liraglutide, 26 with once-weekly semaglutide, and 79 with once-weekly dulaglutide. The lower number of patients treated with semaglutide compared to dulaglutide depends on the fact that semaglutide was marketed much later in Puglia (October 2019).

Finally, for the duration of the study, the time trends and mean changes in 19 variables were evaluated: BMI, weight, AC, SBP, DBP, HR, HOMA index, serum levels of fasting blood glucose, HbA1c, creatinine with estimated glomerular filtration rate (eGFR), uric acid, total cholesterol, low density lipoprotein (LDL)-cholesterol, high density lipoprotein (HDL)-cholesterol, triglycerides, alanine aminotransferase (ALT), aspartate aminotransferase (AST), and γ-glutamyl transferase (γGT). Total daily International Units (IU) of insulin (SA + LA) were also recorded at each time point.


**Compliance with ethics guidelines:** The study was approved by the Clinical Investigation Ethics Committee of the University of Bari Medical Center (Ethical approval number: PZZ_DM2_2020), and all patients gave written informed consent to take part.

### 2.2 Statistical analysis

Comparisons between groups were made by Student’s *t*-test and Chi-square test for continuous and categorical variables, respectively. Mixed models were applied to assess trends of clinical parameters over time. To evaluate the within-subject covariance matrix, an unstructured within-subject covariance and sandwich estimator for robustness and standard error was employed. Statistical evaluations were performed with Stata software (StataCorp. 2021. Stata Statistical Software: Release 17. College Station, TX: StataCorp, LLC). The statistical significance threshold was set at *p* < 0.05.

## 3 Results

Baseline characteristics of the study population are summarized in Table 1. The wide age range of patients treated with the new antidiabetic drugs at our center is immediately evident. The patients included in the study were mainly males, and had high mean values of BW, AC, and BMI. Like for age, there was a wide variation in these clinical parameters, ranging from normal to clearly pathological values (Table 1). Table 1 also shows that the baseline HbA1c or fasting glycemia were not necessarily high prior to prescribing these drugs and that inter-individual variability was very evident also with reference to renal function parameters. Since variable degrees of resistance to insulin action were expected in these patients, we also evaluated the main indirect indicators of insulin resistance with the HOMA index, and pancreatic function reserve by assaying serum C-peptide. As shown in Table 1, the HOMA index was actually high in the study population but a wide range of variation was observed in both HOMA index values and C-peptide serum levels. Vitamin D dosage was available in 108/167 patients and mean levels were found to be low; of note, only 18/108 patients (16.7%) exhibited normal vitamin D values. Finally, Table 1 indicates the mean total daily dose of exogenous insulin (short acting [SA] and/or long acting [LA] insulin) used at baseline by 39 of the 167 (23.4%) patients. As to other antidiabetic drugs, most patients (146/167, 87.4%) were on metformin therapy, alone or in association with other molecules, while only 9/167 (5.4%) were taking no antidiabetic therapy at baseline. With the exception of metformin, all oral antidiabetic drugs previously used by the study population had been discontinued at the time of prescribing a SGLT2i or a GLP1RA.

**Table 1:** Baseline characteristics of the total study population (*n* = 167)

Variables	Values	Minimum value	Maximum value
	(mean value ± SD)		
Age (years)	62.9 ± 9.5	38	83
Gender, male/female [*n* (%)]	113/54 (67.7/32.3%)		
Weight (kg)	91.5 ± 18.2	52	170
BMI (kg/m^2^)	32.7 ± 6.1	21.6	60.9
Abdominal circumference (cm)	111.2 ± 13.9	74	154
SBP (mmHg)	129.2 ± 16.4	90	180
DBP (mmHg)	76.3 ± 10.5	50	110
HR (bpm)	72.4 ± 11.0	46	120
Fasting glucose (mg/dl)	158.6 ± 52.6	87	361
HbA1c (mmol/mol)	62.4 ± 18.1	30	116
C-peptide (ng/ml)	2.9 ± 1.6	0.8	10.9
HOMA index (*n* = 84)	5.0 ± 3.9	0.3	21.9
Vitamin D (ng/ml) (*n* = 108)	22.7 ± 13.4	3	68.5
Creatinine (mg/dl)	0.93 ± 0.31	0.40	2.01
e-GFR (ml/min)	83.3 ± 20.4	26	118
BUN (mg/dl)	42.6 ± 13.1	15	93
Uric acid (mg/dl)	5.4 ± 1.7	1.7	11.3
Cholesterol (mg/dl)	146.8 ± 37.1	25	335
LDL-cholesterol (mg/dl)	75.8 ± 30.7	24	241
HDL-cholesterol (mg/dl)	45.4 ± 13.4	20	116
Triglycerides (mg/ml)	146.2 ± 93.7	46	963
AST (U/L)	24.9 ± 13.6	12	76
ALT (U/L)	34.2 ± 24.5	8	156
γ-GT (U/L)	43.0 ± 38.0	7	296
U-ACR (mg/g) (*n* = 104)	62.3 ± 177.1	0	1,330
SA + LA Insulin (IU) (*n* = 39)	36.9 ± 25.4	10	120

Data are presented as mean value ± SD or as frequency and percentage.

**Abbreviations**: BMI: body mass index; SBP: systolic blood pressure; DBP: diastolic blood pressure; HbA1c: glycated hemoglobin; HOMA index: fasting glucose x fasting insulin/405; e-GFR: estimated-glomerular filtration rate; BUN: blood urea nitrogen; LDL: low density lipoprotein; HDL: high density lipoprotein; AST: aspartate aminotransferase; ALT: alanine aminotransferase; γ-GT: gamma-glutamyl transferase; U-ACR: urine albumin-to-creatinine Ratio; SA: short acting; LA: long acting; IU: international units.

Table 2 summarizes and compares the characteristics of the patients in the 2 groups, subdivided according to the prescribed drug, SGLT2i (Group 1, *n* = 54) or GLP1RA (Group 2, *n* = 113). As illustrated in Table 2, the patients belonging to the GLP1RA group exhibited significantly higher values of both BMI and AC, while Group 1 patients had higher fasting glucose and HbA1c levels. A significant difference was also observed as regards the renal function parameters. It must be considered that throughout the study period, patients with eGFR values <60 ml/min at T0 were not eligible for treatment with SGLT2i in Italy. Consequently, patients treated with this drug had significantly higher eGFR values and lower creatinine levels as compared with the other group (Table 2). The average daily insulin IU (SA and/or LA) per patient were comparable in the two groups. Finally, 22 out of 54 patients (40.7%) for whom SGLT2i therapy was preferred had a previous major CV event in the clinical history, 7/22 patients having suffered dilated cardiomyopathy. Of note, a lower number of patients (26/113, 23.0%) in the GLP1RA group had already experienced a major CV event, and only 1 patient suffered from dilated cardiomyopathy.

**Table 2:** Baseline characteristics of the study population divided according to the drug prescribed

Variables	SGLT2i	GLP1RA	Significance
	(*n* = 54)	(*n* = 113)	
Age (years)	61.2 ± 10.0	63.7 ± 9.1	*p* = 0.110
Gender, male [*n* (%)]	37 (68.5)	76 (67.3)	*p* = 0.870
Weight (kg)	89.0 ± 20.2	92.7 ± 17.1	*p* = 0.226
BMI (kg/m^2^)	31.1 ± 5.4	33.5 ± 6.3	***p*** **= 0.017**
Abdominal circumference (cm)	107.5 ± 15.1	112.9 ± 13.0	***p*** **= 0.021**
SBP (mmHg)	127.6 ± 16.4	130.0 ± 16.5	*p* = 0.405
DBP (mmHg)	76.0 ± 12.1	76.4 ± 9.9	*p* = 0.804
Heart rate	71.9 ± 13.0	72.7 ± 10.1	*p* = 0.672
Fasting glucose (mg/dl)	187.6 ± 65.6	144.0 ± 37.9	***p*** **< 0.001**
HbA1c (mmol/mol)	71.8 ± 21.0	57.8 ± 14.4	***p*** **< 0.001**
C-peptide (ng/ml)	3.0 ± 2.0	2.9 ± 1.3	*p* = 0.704
HOMA index (*n* = 84)	5.3 ± 3.9	4.8 ± 3.9	*p* = 0.587
Vitamin D (ng/ml) (*n* = 108)	19.6 ± 13.6	24.6 ± 13.1	*p* = 0.062
Creatinine (mg/dl)	0.85 ± 0.25	0.97 ± 0.33	***p*** **= 0.025**
e-GFR (ml/min)	90.5 ± 16.7	79.6 ± 21.2	***p*** **= 0.002**
BUN (mg/dl)	40.7 ± 10.9	43.6 ± 14.2	*p* = 0.240
Uric acid (mg/dl)	5.3 ± 1.8	5.4 ± 1.7	*p* = 0.633
Cholesterol (mg/dl)	149.9 ± 34.6	145.3 ± 38.3	*p* = 0.469
LDL-cholesterol (mg/dl)	77.5 ± 29.8	75.0 ± 31.2	*p* = 0.644
HDL-cholesterol (mg/dl)	44.5 ± 12.6	45.8 ± 13.8	*p* = 0.555
Triglycerides (mg/ml)	165.6 ± 138.0	136.9 ± 60.8	*p* = 0.070
AST (U/L)	23.5 ± 14.0	25.7 ± 13.3	*p* = 0.353
ALT (U/L)	32.1 ± 23.0	35.4 ± 25.3	*p* = 0.442
γ-GT (U/L)	39.1 ± 29.9	45.3 ± 41.9	*p* = 0.365
U-ACR (mg/g) (*n* = 104)	103.5 ± 282.7	44.0 ± 97.6	*p* = 0.115
SA + LA insulin (IU) (*n* = 39)	38.7 ± 23.0 (*n* = 16)	35.7 ± 26.9 (*n* = 23)	*p* = 0.399

Data are presented as mean value ± SD or as frequency and percentage. Statistically significant *p* values (<0.05) are shown in bold.

**Abbreviations:** BMI: body mass index; SBP: systolic blood pressure; DBP: diastolic blood pressure; HbA1c: glycated hemoglobin; HOMA index: fasting glucose × fasting insulin/405; e-GFR: glomerular filtration rate; BUN: blood urea nitrogen; LDL: low density lipoprotein; HDL: high density lipoprotein; AST: aspartate aminotransferase; ALT: alanine aminotransferase; γ-GT: gamma-glutamyl transferase; U-ACR: urine albumin-to-creatinine ratio; SA: short acting; LA: long acting; IU: international units.

Tables 3 and 4 summarize the time trends of the 19 parameters of interest as compared to baseline at each time point (T3, T6, and T12) during therapy with SGLT2i or GLP1RA. The different basal characteristics of the 2 groups of patients led us to consider it more appropriate to analyze changes over time within each group and not between groups. Therefore, the effects of the 2 drug classes are described but not statistically compared with each other. The time analysis highlighted that BW, BMI, and AC progressively improved with both SGLT2i and GLP1RA throughout the observation period. In particular, in the SGLT2i group, a significant BW reduction was already evident after 3 months, BMI after 6 months, and AC after 12 months of therapy (Table 3), while in the GLP1RA group, all parameters were found to be significantly improved after just 3 months of therapy and showed further improvements during the 12 month follow-up (Table 4).

**Table 3:** Time trends of clinical parameters in patients treated with SGLT2i

Outcome	Months	Estimated mean values (95% CI)	Estimated mean change from baseline (95% CI)	*P* ^(a)^
Weight (kg)	0	88.4 (83.9–93.0)		
	3	85.7 (81.0–90.3)	−2.8 (−4.4 to –1.1)	**0.001**
	6	86.1 (81.5–90.7)	−2.3 (−3.9 to –0.7)	**0.004**
	12	85.0 (80.4–89.6)	−3.4 (−5.2 to –1.7)	**<0.001**
BMI	0	31.0 (29.5–32.5)		
	3	30.3 (28.8–31.9)	−0.7 (−1.4 to –0.1)	0.069
	6	30.3 (28.8–31.8)	−0.7 (−1.4 to –0.1)	**0.04**
	12	29.9 (28.3–31.4)	−1.1 (−1.9 to –0.4)	**0.004**
AC (cm)	0	107.9 (104.2–111.6)		
	3	107.0 (103.0–111.0)	−0.9 (−3.5 to 1.7)	0.5
	6	108.9 (105.1–112.8)	1.0 (−1.4 to 3.5)	0.4
	12	105.2 (101.2–109.1)	−2.72 (−5.3 to –0.1)	**0.039**
SBP (mmHg)	0	127.8 (123.5–132.2)		
	3	118.2 (112.8–123.5)	−9.7 (−15.1 to –4.2)	**<0.001**
	6	124.5 (119.6–129.4)	−3.3 (−8.3 to 1.6)	0.184
	12	123.1 (117.9–128.2)	−4.7 (−10.0 to 0.5)	0.077
DBP (mmHg)	0	75.8 (73.0–78.7)		
	3	70.2 (66.5–73.9)	−5.6 (−9.6 to –1.6)	0.006
	6	74.1 (70.8–77.4)	−1.7 (−5.4 to 1.9)	0.353
	12	72.5 (69.0–76.0)	−3.3 (−7.2 to 0.5)	0.09
HR (bpm)	0	71.5 (68.4–74.6)		
	3	68.9 (64.2–73.6)	−2.6 (−7.3 to 2.1)	0.277
	6	69.9 (66.4–73.5)	−1.5 (−5.0 to 2.0)	0.387
	12	70.3 (66.8–73.8)	−1.2 (−4.7 to 2.3)	0.512
Fasting glucose (mg/dl)	0	186.4 (173.0–199.7)		
	3	143.3 (127.8–158.8)	−43.1 (−59.6 to –26.6)	**<0.001**
	6	141.7 (127.0–156.4)	−44.6 (−60.4 to –28.8)	**<0.001**
	12	144.4 (128.3–160.5)	−42.0 (−59.1 to –24.9)	**<0.001**
HbA1c (mmol/mol)	0	71.3 (66.9–75.8)		
	3	62.3 (57.2–67.4)	−9.0 (−14.1 to –4.0)	**<0.001**
	6	60.6 (55.7–65.6)	−10.7 (−15.6 to -5.8)	**<0.001**
	12	60.8 (55.6–66.0)	−10.5 (−15.7 to –5.3)	**<0.001**
HOMA index	0	5.4 (4.3–6.5)		
	3	2.9 (0.9–4.9)	−2.5 (−4.7 to –0.2)	**0.03**
	6	3.0 (1.7–4.4)	−2.4 (−4.0 to –0.7)	**0.005**
	12	2.5 (1.2–3.7)	−2.9 (−4.4 to –1.4)	**<0.001**
Creatinine (mg/dl)	0	0.86 (0.80–0.91)		
	3	0.90 (0.83–0.96)	0.04 (−0.02 to 0.10)	0.187
	6	0.89 (0.82–0.95)	0.03 (−0.03 to 0.08)	0.342
	12	0.88 (0.81–0.94)	0.02 (−0.04 to 0.08)	0.534
e-GFR (ml/min)	0	89.8 (85.9–93.7)		
	3	85.1 (80.6–89.6)	−4.7 (−9.0 to –0.3)	**0.035**
	6	87.4 (82.9–91.9)	−2.4 (−6.6 to 1.9)	0.271
	12	88.5 (83.9–93.1)	−1.3 (−5.7 to 3.1)	0.565
Uric acid (mg/dl)	0	5.3 (4.9–5.7)		
	3	4.5 (3.9–5.0)	−0.8 (−1.4 to –0.2)	**0.005**
	6	4.4 (3.9–4.8)	−0.9 (−1.4 to –0.4)	**<0.001**
	12	4.3 (3.8–4.7)	−1.0 (−1.5 to –0.5)	**<0.001**
Cholesterol (mg/dl)	0	148.5 (139.7–157.3)		
	3	148.8 (135.9–161.7)	0.3 (−13.6 to 14.3)	0.964
	6	139.3 (128.6–149.9)	−9.2 (−21.1 to 2.7)	0.131
	12	135.0 (123.7–146.2)	−13.5 (−26.0 to –1.0)	**0.034**
LDL-cholesterol (mg/dl)	0	76.5 (69.5–83.6)		
	3	72.1 (60.8–83.4)	−4.4 (−16.5 to 7.7)	0.477
	6	68.4 (59.6–77.2)	−8.1 (−18.0 to 1.8)	0.107
	12	63.6 (54.7–72.5)	−12.9 (−23.0 to –2.9)	**0.012**
HDL-cholesterol (mg/dl)	0	45.2 (42.2–48.1)		
	3	46.6 (43.1–50.1)	1.4 (−1.1 to 4.0)	0.253
	6	45.6 (42.3–48.8)	0.4 (−1.8 to 2.6)	0.723
	12	47.1 (43.8–50.4)	1.9 (−0.3 to 4.2)	0.092
Triglycerides (mg/dl)	0	162.4 (126.7–198.0)		
	3	204.2 (153.4–255.1)	41.9 (−11.2 to 95.0)	0.122
	6	156.8 (115.0–198.7)	−5.5 (−50.5 to 39.4)	0.809
	12	134.0 (89.4–178.6)	−28.3 (−75.8 to 19.1)	0.241
AST (U/L)	0	23.0 (18.1–28.0)		
	3	33.3 (24.6–42.0)	10.3 (1.2 to 19.4)	**0.027**
	6	24.0 (17.0–31.1)	1.0 (−6.6 to 8.6)	0.794
	12	22.4 (15.7–29.1)	−0.6 (−7.8 to 6.6)	0.873
ALT (U/L)	0	31.3 (24.4–38.1)		
	3	40.8 (29.8–51.8)	9.6 (−1.2 to 20.4)	0.083
	6	30.6 (21.4–39.8)	−0.7 (−9.6 to 8.3)	0.881
	12	31.8 (23.0–40.6)	0.5 (−8.0 to 9.0)	0.904
γ-GT (U/L)	0	38.0 (27.8–48.2)		
	3	46.8 (28.6–65.1)	8.9 (−9.7 to 27.5)	0.351
	6	41.5 (26.4–56.7)	3.5 (−12.1 to 19.2)	0.657
	12	31.8 (18.9–44.6)	−6.2 (−19.6 to 7.2)	0.363

Data are presented as mean value; Confidence Interval (CI) is also indicated.

**Notes:** (a): statistical significance versus baseline values. Statistically significant *p* values (<0.05) are shown in bold.

**Abbreviations:** BMI: body mass index; AC: abdominal circumference; SBP: systolic blood pressure; DBP: diastolic blood pressure; HR: heart rate; HbA1c: glycated hemoglobin; HOMA index: fasting glucose × fasting insulin/405; e-GFR: estimated-glomerular filtration rate; LDL: low density lipoprotein; HDL: high density lipoprotein; AST: aspartate aminotransferase; ALT: alanine aminotransferase; γ−GT: gamma-glutamyl transferase.

**Table 4:** Time trends of clinical parameters in patients treated with GLP1RA

Outcome	Months	Estimated mean values (95% CI)	Estimated mean change from baseline (95% CI)	*p* ^(a)^
Weight (kg)	0	92.5 (89.6–95.3)		
	3	88.7 (85.7–91.7)	−3.8 (−4.9 to –2.5)	**<0.001**
	6	87.7 (84.8–90.6)	−4.8 (−5.8 to –3.7)	**<0.001**
	12	87.4 (84.4–90.3)	−5.1 (−6.3 to –3.9)	**<0.001**
BMI (kg/m^2^)	0	33.4 (32.3–34.4)		
	3	32.0 (30.9–33.1)	−1.4 (−1.8 to –1.0)	**<0.001**
	6	31.6 (30.5–32.7)	−1.8 (−2.2 to –1.4)	**<0.001**
	12	31.5 (30.4–32.5)	−1.9 (−2.3 to –1.5)	**<0.001**
AC (cm)	0	112.7 (110.4–115.1)		
	3	110.5 (107.8–113.1)	−2.3 (−3.9 to –0.7)	**0.005**
	6	110.3 (107.8–112.7)	−2.4 (−3.8 to –1.1)	**<0.001**
	12	109.3 (106.8–111.9)	−3.4 (−4.8 to –1.9)	**<0.001**
SBP (mmHg)	0	130.0 (127.0–132.9)		
	3	128.4 (124.1–132.7)	−1.5 (−6.1 to 3.1)	0.511
	6	125.1 (121.6–128.7)	−4.9 (−8.7 to −0.9)	**0.016**
	12	127.7 (123.9–131.5)	−2.3 (−6.4 to 1.9)	0.288
DBP (mmHg)	0	76.3 (74.5–78.0)		
	3	75.7 (73.1–78.3)	−0.6 (−3.4 to −2.2)	0.676
	6	73.6 (71.5–75.8)	−2.7 (−5.0 to −0.2)	**0.031**
	12	75.5 (73.2–77.8)	−0.8 (−3.3 to 1.8)	0.568
HR (bpm)	0	72.7 (70.8–74.6)		
	3	74.9 (72.0–77.7)	−2.1 (−0.8 to 5.1)	0.159
	6	74.4 (71.9–76.9)	1.7 (−0.9 to 4.3)	0.200
	12	75.4 (72.9–77.9)	2.7 (0.0–5.3)	**0.048**
Fasting glucose (mg/dl)	0	144.3 (137.2–151.3)		
	3	132.4 (123.3–141.4)	−11.9 (−20.8 to −2.9)	**0.009**
	6	125.4 (117.4–133.3)	−18.9 (−26.7 to −11.1)	**<0.001**
	12	122.5 (113.8–131.2)	−21.8 (−30.3 to −13.2)	**<0.001**
HbA1c (mmol/mol)	0	57.7 (55.3–60.1)		
	3	48.3 (45.4–51.2)	−9.3 (−11.8 to −6.8)	**<0.001**
	6	48.0 (45.3–50.6)	−9.7 (−11.9 to −7.5)	**<0.001**
	12	48.9 (46.1–51.8)	−8.7 (−11.1 to −6.3)	**<0.001**
HOMA index	0	4.9 (4.1–5.7)		
	3	4.3 (2.8–5.9)	−0.6 (−2.2 to 1.1)	0.508
	6	4.4 (3.3–5.5)	−0.5 (−1.7 to 0.7)	0.426
	12	4.2 (3.2–5.2)	−0.7 (−1.8 to 0.4)	0.206
Creatinine (mg/dl)	0	0.98 (0.92–1.03)		
	3	1.02 (0.96–1.08)	0.04 (0.00–0.09)	0.061
	6	1.02 (0.96–1.08)	0.04 (0.00–0.08)	0.062
	12	0.94 (0.88–1.00)	−0.04 (−0.08–0.01)	0.109
e-GFR (ml/min)	0	79.2 (75.9–82.5)		
	3	78.6 (74.6–82.5)	−0.6 (−3.9 to 2.6)	0.697
	6	77.5 (73.8–81.3)	−1.7 (−4.7 to 1.3)	0.269
	12	81.6 (77.9–85.4)	2.4 (−0.7 to 5.4)	0.126
Uric acid (mg/dl)	0	5.4 (4.4–6.3)		
	3	5.3 (4.3–6.4)	0.0 (−1.4 to 1.4)	0.976
	6	5.4 (4.3–6.5)	0.0 (−1.3 to 1.4)	0.969
	12	5.4 (4.2–6.6)	0.0 (−1.4 to 1.4)	0.986
Cholesterol (mg/dl)	0	144.8 (138.3–151.3)		
	3	137.8 (128.6–147.1)	−7.0 (−16.4 to 2.4)	0.147
	6	139.6 (131.9–147.2)	−5.2 (−13.1 to 2.6)	0.191
	12	139.7 (131.6–147.9)	−5.1 (−13.4 to 3.2)	0.232
LDL-cholesterol (mg/dl)	0	74.9 (69.4–80.4)		
	3	72.6 (64.6–80.6)	−2.3 (−10.6 to 5.9)	0.581
	6	69.9 (63.3–76.5)	−5.0 (−11.9 to 1.9)	0.158
	12	67.8 (60.8–74.8)	−7.1 (−14.4 to 0.2)	0.058
HDL-cholesterol (mg/dl)	0	45.5 (43.2–47.7)		
	3	44.2 (41.5–47.0)	−1.2 (−3.4 to 0.9)	0.272
	6	46.8 (44.3–49.3)	1.3 (−0.4 to 3.1)	0.138
	12	46.9 (44.3–49.4)	1.4 (−0.5 to 3.3)	0.141
Triglycerides (mg/dl)	0	136.9 (124.4–149.5)		
	3	133.6 (116.1–151.0)	−3.4 (−20.2 to 13.4)	0.694
	6	128.3 (113.7–142.9)	−8.6 (−22.4 to 5.2)	0.220
	12	136.6 (121.1–152.0)	−0.4 (−15.1 to 14.4)	0.961
AST (U/L)	0	25.8 (23.7–27.8)		
	3	20.8 (17.4–24.2)	−4.9 (−8.6 to −1.2)	**0.009**
	6	19.9 (17.2–22.5)	−5.9 (−8.9 to −2.9)	**<0.001**
	12	20.5 (17.8–23.3)	−5.2 (−8.3 to −2.1)	**0.001**
ALT (U/L)	0	35.5 (31.7–39.3)		
	3	26.6 (20.2–33.0)	−8.9 (−16.1 to −1.7)	**0.015**
	6	24.0 (19.0–29.0)	−11.5 (−17.5 to −5.6)	**<0.001**
	12	28.6 (23.5–33.7)	−6.9 (−13.0 to −0.9)	**0.025**
γ-GT (U/L)	0	45.5 (38.3–52.7)		
	3	35.4 (23.9–46.9)	−10.1 (−22.1 to 2.0)	**0.101**
	6	35.5 (26.5–44.6)	−9.9 (−19.7 to −0.1)	**0.047**
	12	32.1 (22.8–41.3)	−13.4 (−23.2 to −3.5)	**0.008**

Data are presented as mean value; Confidence Interval (CI) is also indicated.

**Notes:** (a): statistical significance versus baseline values. Statistically significant *p* values (<0.05) are shown in bold.

**Abbreviations:** BMI: body mass index; AC: abdominal circumference; SBP: systolic blood pressure; DBP: diastolic blood pressure; HR: heart rate; HbA1c: glycated hemoglobin; HOMA index: fasting glucose x fasting insulin/405; e-GFR: estimated-glomerular filtration rate; LDL: low density lipoprotein; HDL: high density lipoprotein; AST: aspartate aminotransferase; ALT: alanine aminotransferase; γ-GT: gamma-glutamyl transferase.

Both therapeutic regimens induced a transient reduction in SBP and DBP, although this was significant only at T3 (for SGLT2i) and T6 (for GLP1RA). The heart rate was affected only by GLP1RA, showing a significant increase, but not before 12 months of therapy (Table 4).

In both the subsets, the decrease in glucose levels compared to baseline was found to be highly significant already at T3, as well as after 6 and 12 months of therapy (Tables 3 and 4). Although the HbA1c parameter is not susceptible to sudden changes, it is interesting to note that the reduced levels induced by either class of drugs were significant compared to baseline already by the third month of therapy, and remained so up to T12 (Tables 3 and 4). As is well known, the absolute changes in both fasting blood glucose and HbA1c were proportional to their respective starting values.

The HOMA index underwent a significant and progressive decrease from T3 to T12, selectively as an effect of SGLT2i therapy (Table 3), while the GLP1RA-related reduction in the index values was negligible throughout the observation period (Table 4). As regards the renal function parameters, the mean changes in serum creatinine over the 12 months never reached statistical significance in the study population, regardless of the drug used. Also, eGFR values remained almost unchanged at each follow-up, in both GLP1RA (Table 4) and SGLT2i (Table 3) treated patients, with the exception of a transient reduction at T3 in the latter group (Table 3).

The two therapeutic regimens elicited divergent effects on uric acid and liver enzymes. SGLT2i caused an early, highly significant, and progressive decrease from T3 to T12 in serum uric acid levels, while the drug influence on AST, ALT, and γGT was mild and not significant, except for some evidence of an isolated increase in AST levels at the third month of follow-up (Table 3). On the contrary, GLP1RA did not modify uric acid levels at all, while inducing a notable reduction in both AST and ALT levels at each follow-up time, as well as reducing γGT levels after 6 and 12 months of therapy (Table 4). Finally, only SGLT2i demonstrated the ability to partially improve the lipid profile, significantly and selectively reducing serum levels of total and LDL cholesterol, but not of triglycerides, after 12 months of therapy (Table 3). Unfortunately, although of great clinical interest, it was not possible to assess the urine albumin-to-creatinine ratio (U-ACR) data during the follow-up in an adequate number of patients from either group, so the time trend analysis of this parameter could not be included in the study.

The changes in insulin therapy deserve special mention. In group 2, 12 patients treated with SA insulin regimens were informed that GLP1RA should replace insulin administration, because the reimbursement of this pharmacological association was not provided for by the Italian National Health System. It should be stressed that they had been taking SA insulin at doses ranging from 10 to 90 IU/day; in total, 372 IU of SA insulin were discontinued in this group already at T0. More gradual but still considerable changes in the use of LA insulin occurred in the same group. In fact, after 1 year of GLP1RA therapy, 9/23 (39.1%) insulin-treated patients had completely suspended any type of insulin and the others had significantly reduced overall daily insulin consumption, so that in total, 579 IU of insulin (SA + LA) were ultimately discontinued in group 2 patients. In group 1, SA insulin administration was suspended at T0 in 3 of 6 SA insulin-treated patients. During the follow-up period, the complete withdrawal of SA and/or LA insulin was achieved in 4/16 patients (25%) and, as a final result of SGLT2i therapy, 315 IU of insulin (SA + LA) were discontinued.

There were 13/167 (7.8%) cases of dropout in the entire population under study. We found that the therapy had been suspended prematurely in 4/113 (3.5%) patients in group 2 due to poor glyco-metabolic control; unexpectedly, none of the patients discontinued treatment due to gastro-intestinal effects of GLP1RA. Instead, 9/54 SGLT2i-treated patients (16.7%) stopped therapy earlier than expected due to: i) poor compliance to any therapy (1 patient); ii) worsening of renal function parameters (1 patient); iii) unsatisfactory glyco-metabolic control (6 patients, 2 of whom complained of increased appetite); and iv) lack of weight loss despite the achievement of a satisfactory glyco-metabolic control (1 patient).

## 4 Discussion

The advent of SGLT2i and GLP1RA has radically changed the guidelines of diabetes therapy [2,4]. Based on the results of the latest clinical trials, the new standards for T2D are no longer focused only on reducing HbA1c and minimizing hypoglycemic risk, but are aimed at reducing CVR and micro- and macrovascular adverse complications as well as global mortality of the diabetic patient. However, the RCTs inclusion criteria are known to be very selective and preclude the recruitment of “complex” patients, such as the elderly, patients with chronic kidney disease, or suffering from multiple comorbidities. Thus, when dealing with diabetic patients, real life studies are particularly interesting to evaluate treatment effectiveness in everyday life, thereby integrating RCTs, which are of course, irreplaceable. In the current study, we retrospectively analyzed data on a population of diabetic patients attending our Metabolic Disorders Outpatients Clinic in Bari, who had been treated under routine care with either a SGLT2i or a GLP1RA. First of all, the wide age range of the patients included in the study (38–83 years) indicates the extreme manageability of these two classes of drugs, that can be safely used even in older age groups. This characteristic, primarily attributable to the minimization of the hypoglycemic risk guaranteed by both categories, is one of the most important features of these pharmacological approaches. Patients were mostly males, with a slightly higher male to female ratio than in the epidemiological data in literature. The study population was heterogeneous also in terms of biometric parameters because despite the SGLT2i and GLP1RA ability to induce a significant and lasting decrease in weight, we did not use them exclusively in overweight or obese subjects. In line with this trend, the latest data provided by RCTs increasingly support the idea that the broad spectrum of biological and metabolic effects of these new antidiabetic agents makes them suitable for the treatment of normal weight patients as well. Thus, although our patients were mostly affected by abdominal obesity, the study also included subjects with normal weight, BMI, and AC values (minimum values: 52 kg, 21.6 kg/m^2^, and 74 cm, respectively). Similarly, our data highlight the fact that these drugs had been prescribed even in patients with normal fasting glycemia and HbA1c values (minimum values: 87 mg/dl and 30 mmol/mol, respectively), as is now suggested by the growing evidence emerging from RCTs. In these trials, both SGLT2i and GLP1RA were shown to provide such effective cardio- and nephron-protection that their use was finally recommended regardless of HbA1c values [4,8]. This was of particular importance in Italy, where this recommendation was issued by AIFA (*Agenzia Italiana del Farmaco*) only in July 2018; until then, GLP1RA could be reimbursed only within an HbA1c range of 53–75 mmol/mol. In particular, the REWIND study played a key role in modifying this prescription indication [9]. For the first time, this trial provided evidence of a GLP1RA-mediated “cardio-preventive effect,” demonstrating the efficacy of once-weekly dulaglutide in primary prevention, in terms of a reduced incidence of all CV outcomes [9]. Previously, both the LEADER and SUSTAIN-6 trials had shown superior results on CV outcomes of liraglutide and semaglutide versus *placebo* but most of the enrolled patients were undergoing secondary prevention [10,11].

Assessment of the baseline renal function of the study population highlighted, once again, a great inter-individual variability. In particular, since the safe use of most GLP1RA had been progressively extended up to eGFR values of 15 ml/min, patients with an impaired renal function could be treated with new antidiabetic drugs (minimum eGFR: 26 ml/min).

Routine determination of the pancreatic β-cell reserve has become essential in daily clinical practice for an optimal therapeutic management of diabetic patients. Inappropriate or overuse of exogenous insulin in dysmetabolic and high CVR patients is no longer acceptable and must be considered deleterious to their health and quality of life [12]. Accordingly, we usually perform a baseline assessment of the metabolic status of T2D patients, evaluating the main indirect indicators of insulin resistance and pancreatic function reserve, with the HOMA index and C-peptide, respectively. In particular, since C-peptide serum levels are not significantly affected by exogenous insulin administration, they are particularly relevant in the metabolic evaluation of patients on insulin therapy [13], in whom insulinemia cannot be used for the HOMA index calculation. Therefore, the C-peptide assay may be a simple test to identify subjects whose residual β-cell function is still sufficient to indicate the use of the new antidiabetic drugs, so as to delay the administration of exogenous insulin and progressively reduce or completely suspend unnecessary insulin therapy [12]. Notably, the evidence in our population of minimum C-peptide values within the normal range indicated a preserved β-cell ability to produce insulin in all these patients. Therefore, it seemed rational to significantly reduce the total amount of insulin globally administered to 39 of the 167 patients included in this study.

Our group had already shown that patients with metabolic syndrome have low serum levels of vitamin D [14,15]; the diabetic patients included in this study exhibited similarly low levels of the vitamin.

The comparison of the patients’ baseline characteristics after their subdivision into group 1 and group 2 (SGLT2i- and GLP1RA-treated patients, respectively) highlighted that patients belonging to the GLP1RA group had higher baseline values of BMI and AC. In this regard, a decreased appetite is a “useful side effect” that selectively characterizes GLP1RA treatment, due to the slowing of gastric emptying and an increased sense of satiety mediated by a central effect [16,17]. Conversely, despite the calorie loss/energy deficit (300 kcal/day) induced by SGLT2i therapy, this pharmacological approach is not always associated with the expected weight loss, likely as a result of “compensatory hyperphagia” and changes in energy expenditure aimed at attenuating this energy imbalance [18]. These characteristics likely led to a preference for GLP1RA when achieving a reduction in BW was a key treatment goal for the patient.

On the other hand, group 1 patients exhibited higher mean baseline values of fasting glucose and HbA1c as compared to group 2, which appears reasonable if we consider that the increased renal elimination of glucose induced by this drug class is an effective and rapid mechanism for obtaining adequate glycemic compensation. At the same time, poor glyco-metabolic control did not allow the complete discontinuation of SA insulin therapy at T0 in these patients, so making them ineligible for GLP1RA prescription, in accordance with Italian standards. These aspects contributed to influence the choice of SGLT2i that, in line with the literature data, were also the preferred therapy for those of our diabetic patients suffering from dilated cardiomyopathy.

Finally, the difference between the 2 groups as regards renal function parameters was not surprising, in view of the Italian SGLT2i prescribing limits when the study started. In fact, in Italy, with baseline eGFR values below 60 ml/min, patients were not eligible for treatment with SGLT2i until June 2020, so eGFR was a discriminating factor in the choice of therapy to be prescribed to our patients before that time. The current situation is different. There is no doubt that SGLT2i required a globally preserved renal function in order to ensure adequate glycemic control, due to their peculiar mechanism of action and glycosuric effect. Nevertheless, the EMPA-REG OUTCOME (for empagliflozin) [19], CANVAS (for canagliflozin) [20], and DECLARE-TIMI58 (dapagliflozin) [21] trials highlighted such significant benefits in terms of composite renal outcomes that nephrologists became increasingly prone to make SGLT2i their first-choice drugs in diabetic patients with evidence of initial kidney damage (microalbuminuria or renal hyperfiltration). More recently, the CREDENCE trial [22] and the DAPA-CKD [23], specifically designed to evaluate canagliflozin and dapagliflozin effects on renal outcomes, respectively, included patients with wide ranges of albuminuria (20–5,000 mg/day) and eGFR (25–90 ml/min). The results of these trials contributed to extend the prescription of these molecules to patients with eGFR values as low as 30 ml/min.

The next step was to analyze the effects of SGLTi or GLP1RA treatment in our real life setting, over a 1 year follow-up period. Our data fully confirmed the main results of RCTs. What is immediately evident from the time trend of the variables of our interest is the significant action of both therapeutic approaches on biometric parameters, with GLP1RA effects being already significant by the third month. Furthermore, an improvement in the patients’ body composition and visceral fat mass, although not directly assessed, can be presumed on the basis of the significant AC reduction induced by both drugs (mean decrease at T12 of 2.7 and 3.4 cm in groups 1 and 2, respectively).

The increase in heart rate as a result of GLP1RA administration was expected, but it did not lead to drug discontinuation in any patient and was found to be significant only after 1 year of therapy.

In line with RCTs, the SGLT2i- and GLP1RA-induced improvement in glycemic levels and glycated Hb values were very satisfactory, despite the complexity and heterogeneity of our study population. These positive effects were already significant after 3 months of treatment and persisted up to T12. The differences observed between the 2 groups were mostly attributable to the different baseline metabolic conditions of patients.

The significant decrease in HOMA index observed over time as a result of SGLT2i therapy is in line with previous *in vitro* studies, showing the normalization of insulin sensitivity induced by phlorizin in animal models [24]. Moreover, it should be considered that SGLT2i-induced glycosuria lowers plasma glucose and insulin levels and raises fasting and post-meal glucagon concentrations. This leads to changes in energy substrate use, fostering the utilization of lipids for energy production and the release of non-esterified fatty acids, which are converted to ketone bodies in the liver through mitochondrial beta oxidation and ketogenesis, resulting in a metabolic condition resembling a prolonged fast [25]. Multiple mechanisms might be involved in this insulin-sensitivity improvement, namely an inhibition of glucose toxicity, increase in adiponectin levels, reduction in visceral fat and lipotoxicity-induced insulin resistance [26,27], the promotion of browning adipose tissue and fat utilization by M2 macrophages [28], and attenuation of inflammatory responses [29,30,31].

The remarkable renal safety of the two therapeutic approaches was suggested by the substantial stability of the renal parameters throughout the study period. Of note, the eGFR value reduction observed in SGLT2i-treated patients, which reached statistical significance only at T3, demanded modification of the therapeutic regimen only in 1 patient, due to his lack of compliance in guaranteeing an adequate water intake.

Finally, the analysis of the last 8 variables evaluated in this study revealed important differences between the 2 classes of antidiabetic drugs. In fact, only SGLT2i were demonstrated to significantly decrease uric acid levels as well as to improve the lipid profile, selectively reducing total and LDL cholesterol serum levels. In light of the well-known ability of these parameters to affect the body’s CVR, a potential contribution of these changes to the SGLT2i-mediated cardioprotection cannot be excluded. Conversely, our finding of a significant decrease in AST, ALT, and γGT levels in GLP1RA-treated patients further supports the beneficial effect of this class on hepatic steatosis [32,33]. In regard to this aspect, it should be borne in mind that important clinical trials are underway to explore the possibility of using GLP1RA in the treatment of Non-alcoholic Fatty Liver Disease (NAFLD) and Non-alcoholic Steatohepatitis (NASH), even in non-diabetic patients [34,35].

The main limitation of this study is ascribable to the prescription limitations of the two classes of drugs existing in Italy in the period analyzed. This inevitably had significant effects on the choice of the drug to be used, regardless of the clinical characteristics of the patients (i.e., non-reimbursement of GLP1RA in association with SA insulin or ineligibility for SGLT2i therapy in patients with eGFR <60 ml/min). These aspects faithfully reflect the problems to be solved in daily clinical practice and the consequent need to adapt. In fact, it is not always possible to prescribe the “best existing therapy” and is still necessary to prescribe the best therapy available for “that” particular patient. Another limitation, also in this case linked to the real-life nature of the study, lies in the heterogeneity of the population analyzed and in the numerical difference between the two groups of patients. Finally, the ability of both SGLT2i and GLP1RA to induce an improvement in body composition in treated patents remains a speculation in this study, but our group is currently conducting a more accurate evaluation of this hypothesis by means of specific investigations.

## 5 Conclusion

Our real-life data clearly confirm the main results obtained with RCTs, which instead recruit highly selected patients to verify the study outcomes. The safety and effectiveness of both SGLT2i and GLP1RA are demonstrated even in diabetic patients with heterogeneous characteristics commonly treated in everyday clinical practice. The use of these drugs is thus strongly recommended, from the perspective of no longer treating just “hyperglycemia” but a complex disease in which the most important goal should be the prevention of CV complications and reduction in overall mortality. For the same reason, the unnecessary use of insulin in patients with a preserved β-cell function should be discouraged, especially when a condition of insulin resistance is present.
